# Supplementary material for: Heat adaptation of phage T7 under an extended genetic code
Source: Virus Evol. 2021 Dec 1;7(2):veab100. doi: 10.1093/ve/veab100 (PMC8923235; doi:10.1093/ve/veab100)
Supplement: veab100_Supp [file veab100_supp.zip › Sequencing_Analysis.zip!/Sequencing_Analysis/A3/evidence/JC_SIDE_1_61.html]

BRESEQ :: Evidence


| New junction evidence | | | | | | | | | | | |
| --- | --- | --- | --- | --- | --- | --- | --- | --- | --- | --- | --- |
|  | | seq id | position | reads (cov) | reads (cov) | score | skew | freq | annotation | gene | product |
| \* | ? | NC\_001604.1 | = 7613 | 2216 (1.270) | 19 (0.010) | 11/586 | 38.5 | 0.93% | intergenic (–/–) | *–/–* | –/– |
|  | ? | NC\_001604.1 | 7912 = | 1900 (1.110) |  |  |  |  | intergenic (–/–) | *–/–* | –/– |
| Rejected: Coverage evenness skew score above cutoff. | | | | | | | | | | | |
| Rejected: Frequency below/above cutoff threshold. | | | | | | | | | | | |

Only 100 of 2220 total aligned reads displayed.  

`CCACGAATATCTCTCGCGCCTTAATGGATGAGTTCACTGAGACAGTAAAAGAGGCCACCCTAAGTCAATGGGGATTCTTTAGCCCATACGGTATTGGCGACAACGATGCTTGTACTATTAACCCTTACGATGGCTGGGCGTGTCAAATTAGCTACATGGAGGAAACACCTGATGGCTCTTTGCGGCACCCATCGTTCGTAATGTTCCGTGGCACCGAGGACAACCCTCAAGAGAAAATGTAATCACACTGGCTCACCTTCGGGTGGGCCTTTCTGCGTTTATAAGGAGACACTTTATGTTTAAGAAGGTTGGTAAATTCCTTGCGGCTTTGGCAGCTATCCTGACGCTTGCGTATATTCTTGCGGTATACCCTCAAGTAGCACTAGTAGTAGTTGGCGCTTGTTACTTAGCGGCAGTGTGTGCTTGCGTGTGGAGTATAGTTAACTGGTAATACGACTCACTAAAGGAGGTACACACCATGATGTACTTAATGCCATTACTCATCGTCATTGTAGGATGCCTTGCGCTCCACTGTAGCGATGATGATATGCCAGATGGTCACGCTTAATACGACTCACTAAAGGAGACACTATATGTTTCG  >  NC_001604.1/7313‑7913`  

                                                                                                                                                                                                                                                                                                            `|`                                                                                                                                                                                                                                                                                                              
    `gAATATCTCTCGCGCCTTAATGGATGAGTTCACTGAGACAGTAAAAGAGGCCACCCTAAGTCAATGGGGATTCTTTAGCCCATACGGTATTGGCGACAACGATGCTTGTACTATTAACCCTTACGATAGCTGGGCGTGTCAAATTAGCTACATGGAGGAAACACCTGATGGCTCTTTGCGGCACCCATCGTTCGTAATGTTCCGTGGCACCGAGGACAACCCTCAAGAGAAAATGTAATCACACTGGCTCACCTTCGGGTGGGCCTTTCTGCGTTTATAAGGAGACACTTTATGTTTaaga                                                                                                                                                                                                                                                                                                          >  1:151501/1‑301 (MQ=255)`  
                  `ccgtAATTGATGAGTTCACTGGAACAGTAAAAGAAGCCACCCTAAGTAAATGGGGATTCTTTAGCCCATACGGTATTGGCGACAACGATGCTTGGACTATTAACCCTTACGATGGCTGGGCGTGTCAAATTAGCTACATGGAGGAAACACCTGATGGCTCTTTGCGGCACCCATCGTTCGTAATGTTCCGTGGCACCGAGGACAACCCCCAAGAGAAAATGTAATCACACTGGCTCACCTTCGGGTGGGCATTTCTGCGTTTATAAGGAGACACTTTATGTTTAAGAAGGTTGGTAAATTc                                                                                                                                                                                                                                                                                            <  1:10623/298‑1 (MQ=255)`  
                               `gTTCACTGAGACAGTAAAAGAGGCCACCCTAAGTCAATGGGGATTCTTTAGCCCATACGGTATTGGCGACAACGATGCTTGTACTATTAACCCTTACGATGGCTGGGCGTGTCAAATTAGCTACATGGAGGAAACACCTGATGGCTCTTTGCGGCACCCATCGTTCGTAATGTTCCGTGGCACCGAGGACAACCCTCAAGAGAAAATGTAATCACACTGGCTCACATTCGGGTGGGCCTTTCTGCGTTTATAAGGAGACACTTTATGTTTAAGAAGGTTGGTAAATTCATTGCGGCTTTgg                                                                                                                                                                                                                                                                               >  1:142888/1‑301 (MQ=255)`  
                               `gTTCACTGAGACAGTAAAAGAGGCCACCCTAAGTCAATGGGGATTCTTTAGCCCATACGGTATTGGCGACAACGATGCTTGTACTATTAACCCTTACGATGGCTGGGAGTGTCAAATTAGCTACATGGAGGAAACACCTGATGGCTCTTTGCGGCACCCATCGTTCGTAATGTTCCGTGGCACCGAGGACAACCCTCAAGAGAAAATGTAATCACACTGGCTCACCTTCGGGTGGGCCTTTCTGCGTTTATAAGGAGACACTTTATGTTTAAGAAGGTTGGTAAATTCCTTGCGGCTTTgg                                                                                                                                                                                                                                                                               >  1:186823/1‑301 (MQ=255)`  
                                         `aCAGTAAAAGAGGCCACCCTAAGTCAATGGGGATTCTTTAGCCCATACGGTATTGGCGACAACGATGCTTGTACTATTAACCCTTACGATGGCTGGGCGTGTCAAATTAGCTACATGGAGGAAACACATGATGGCTCTTTGCGGCACCCATCGTTCGTAATGTTCCGTGGCACCGAGGACAACACTCAAGAGAAAATGTAATCACACTGGCTCACCTTCGGGTGGGCCTTTCTGCGTTTATAAGGAGACACTTTATGTTTAAGAAGGTTGGTAAATTCCTTGCGGcctgtctcttatacac                                                                                                                                                                                                                                                                     >  1:202218/1‑286 (MQ=255)`  
                                          `cAGTAAAAGAGTCCACCCTAAGTCAATGGGGATTCTTTAGCCCATACGGTATTGGCGACAACGATGCTTGTACTATTAACCCTTACGATGGCTGGGCGTGTCAAATTAGCTACATGGAGGAAACACCTGATGGCTCTTTGCGGCACCCATCGTTCGTAATGTTCCGTGGCACCGAGGACAACCCTCAAGAGAAAATGTAATCACACTGGCTCACCTTCGGGTGGGCCTTTCTGCGTTTATAAGGAGACACTTTATGTTTAAGAAGGTTGGTAAATTCCTTGCGGCTTTGGCAGCTATCCTg                                                                                                                                                                                                                                                                    >  1:96025/1‑301 (MQ=255)`  
                                          `cAGTAAAAGAGGCCACCCTAAGTCAATGGGGATTCTTTAGCCCATACGGTATTGGCGACAACGATGCTTGTACTATTAACCCTTACGATGGCTGGGCGTGTCAAATTAGCTACATGGAGGAACCACCTGATGGCTCTTTGCGGCACCCATCGTTCGTAATGTTCCGTGGCACCGAGGACAACCCTCAAGAGAAAATGTAATCACACTGGCTCACCTTCGGGTGGGCCTTTCTGCGTTTATAAGGAGACACTTTATGTTTAAGTAGGTTGGTAAATTCCTTGCGGCTTTGGCAGCTATCCtt                                                                                                                                                                                                                                                                    >  1:281636/1‑300 (MQ=255)`  
                                          `cAGTAAAAGAGGCCACCCTAAGTCAATGGGGATTCGTTAGCCCATACGGTATTGGCGACAACGATGCTTGTACTATTAACCCTTACGATGGCTGGGCGTGTCAAATTAGCTACATGGAGGAAACACCTGATGGCTCTTTGCGGCACCCATCGTTCGTAATGTTCCATGGCACCGAGGACAACCATCAAGAGAAAATGTAATCACACTGGCTCACCTTCGGGTGGGCCTTTCTGCGTTTATAAGGAGACACTTTATGTTTAAGAAGGTTGGTAAATTCCTTGCGGCTTTGGCAGCTATCCTg                                                                                                                                                                                                                                                                    <  1:159037/301‑1 (MQ=255)`  
                                           `aGTAAAAGAGGCCACCCTAAGTCAATGGGGATTCTTTAGCCCATACGGTATTGGCGACAACGATGCTTGTACTATTAACCCTTACGATGGCTGGGCGTGTCAAATTAGCTACATGGAGGAAACACCTGATGGCTCTTTGCGGCACCCATCGTTCGTAATGTTCAGTGGCACCGAGGACAACCCTCAAGAGAAAATGTAATCACACTGGCTCACCTTCGGGTGGGCCTTTCTGCGTTTATAAGGAGACACTTTATGTTTAAGAAGGTTGGTAAATTCCTTGCGGCTTTGGCAGCTATCCTGa                                                                                                                                                                                                                                                                   >  1:174525/1‑301 (MQ=255)`  
                                            `gTAAAAGAGGCCACCCTAAGTCAATGTGGATTCTTTAGCCCATACGGTATTGGCGACAACGATGCTTGTACTATTAACCCTTACGATGGCTGGGCGTGTCAAATTAGCTACATGGAGGAAACACCTGATGGCTCTTTGCGGCACCCATCGTTCGTAATGTTCCGTGGCACCGAGGACAACCCTCAAGAGAAAATGTAATCACACTGGCTCACCTTCGGGTGGGCCTTTCTGCGTTTATAAGGAGACACTTTATGTTTAAGAAGGTTGGTAAATTCCTTGCGGCTTTGGCAGCTATCCTGAc                                                                                                                                                                                                                                                                  >  1:257800/1‑301 (MQ=255)`  
                                            `gTAAAAGAGGCCACCCTAAGTCAATGGGGATTCTTTAGCCCATACGGTATTGGCGACAACGATGCTTGTACTATTAACCCTTACGATGGCTGGGCGTGTCAAATTAGCTACATGGAGGAAACACCTGATGGCTCTTTGCGGCACCCATCGTTCGTAATGTTCCGTGGCACCGAGGACAACCCTCAAGAGAAAATGTAATCACACTGGCTCACCTTCGGGTGGGCCTTTCTGCGTTTATAAGGAGACACTTTATGTTTAAGAAGGTTGGTAAATTCCTTGCGGCTTTGGCAGCTATCCTGAc                                                                                                                                                                                                                                                                  >  1:181138/1‑301 (MQ=255)`  
                                             `aaaaaGAGGCCACCCTAAGTCAATGTGGATTCTTTAGCCCATACGGTATTGGCGACAACGATGCTTGTACTATTAACCCTTACGATGGCTGGGCGTGTCAAATTAGCTACATGGAGGAAACACCTGATGGCTCTTTGCGGCACCCATCGTTCGTAATGTTCCGTGGCACCGATGACAACCCTCAAGAGAAAATGTAATCACACTGGCTCACCTTCGGGTGGGCCTTTCTGCGTTTATAAGGAGACACTTTATGTTTAAGAAGGTTGGTAAATTCCTTGCGGCTTTGGCAGCTATCCTGACg                                                                                                                                                                                                                                                                 <  1:132597/300‑1 (MQ=255)`  
                                                         `cccTAAGTCAATGGGGATTCTTTAGCCCATACGGTATTGGCGACAACGATGCTTGTACTATTAACCCTTACGATGGCTGGGCGTGTCAAATTAGCTACATGGAGGAAACACCTGATGGCTCTTTGCGGCACCCATCGTTCGTAATGTTCCGTGGCACCGAGGACAACCCTCAAGAGAAAATGTAATCACACTGGCTCACCTTCGGGTGGGCCTTTCTGCGTTTATAAGGAGACACTTTATGTTTAAGAAGGTTGGTAAATTCCTTGCGGCTTTGGCAGCTATCCTGACGCTTGCGTATAtt                                                                                                                                                                                                                                                     >  1:277511/1‑301 (MQ=255)`  
                                                         `cccTAAGTCAATGGGGATTCTTTAGACCATACGGTATTGGCGACAACGATGCTTGTACTATTAACCCTTACGATGGCTGGGCGTGTCAAATTAGCTACATGGAGGAAACACCTGATGGCTCTTTGCGGCACCCATCGTTCGTAATGTTCCGTGGCACCGAGGACAACCCTCAAGAGAAAATGTAATCACACTGGCTCACCTTCGGGTGGGCCTTTCTGCGTTTATAAGGAGACACTTTATGTTTAAGAAGGTTGGTAAATTCCTTGCGGCTTTGGCAGCTATCCTGACGCTTGCGTATCtt                                                                                                                                                                                                                                                     >  1:250806/1‑301 (MQ=255)`  
                                                                `tCAATGGGGATTCTTTAGCCCATACGGTATTGGCGACAACGATGCTTGTACTATTAACCCTTACGATGGCTGGGCGTGTCAAATTAGCTACATGGAGGAAACACCTGATGGCTCTTTGCGGCACCCATCGTTCGTAATGTTCCGTGGCACCGAGGACAACCCTCAAGAGAAAATGTAATCACACTGGCTCACCTTCGGGTGGGCCTTTCTGCGTTTATAAGGAGACACTTTATGTTTAAGAAGGTTGGTAAATTCCTTGCGGCTTTGGCAGCTATCCTGACGCTTGCGTATATTCTTGCgg                                                                                                                                                                                                                                              <  1:211220/301‑1 (MQ=255)`  
                                                                   `aTGGGGATTCTTTAGCCCATACGGTATTGGCGACAACGATGCTTGTACTATTAACCCTTACGATGGCTGGGCGTGTCAAATTAGCTACATGGAGGAAACACCTGATGGCTCTTTGCGGCACCCATCGTTCGTAATGTTCCGTGGCACCGAGGACAACCCTCAAGAGAAAATGTAATCACACTGGCTCACCTTCGGGTGGGCCTTTCTGCGTTTATAAGGAGACACTTTATGTTTAAGAAGGTTGGTAAATTCCTTGCGGCTTTGGCAGCTATCCTGACGCTTGCGTATATTCTTGCGGtat                                                                                                                                                                                                                                           >  1:323006/1‑301 (MQ=255)`  
                                                                       `ggATTCTTTAGCCCATACGGTATTGGCGACAACGATGCTTGTACTATTAACCCTTACTATGGCTGGGCGTGTCAAATTAGCTACATGGAGGAAACACCTGATGGCTCTTTGCGGCACCCATCGTTCGTAATGTTCCGTGGCACCGAGGACAACCCTCAAGAGAAAATGTAATCACACTGGCTCACCTTCGGGTGGGCCTTTCTGCGTTTATAAGGAGACACTTTATGTTTAAGAAGGTTGGTAAATTCCTTGCGGCTTTGGCAGCTATCCTGACGCTTGCGTATATTCTTGCGGTATAccc                                                                                                                                                                                                                                       >  1:291778/1‑301 (MQ=255)`  
                                                                       `ggATTCTTTAGCCCATACGGTATTGGCGACAACGATGCTTGTACTATTAACCCTTACGATGGCTGGGCGTGTCAAATTAGCTACATGGAGGAAACACCTGATGGCTCTTTGCGGCACCCATCGTTCGTAATGTTCCGTGGCACCGAGGACAACCCTCAAGAGAAAATGCAATCACACTGGCTCACCTTCGGGTGGGCCTTTCTGCGTTTATAAGGAGACACTTTATGTTTAAGAAGGTTGGTAAATTCCTTGCGGCTTTGGCAGCTATCCTGACGCTTGCGTATATTTTTGCGGTATAccc                                                                                                                                                                                                                                       >  1:247694/1‑301 (MQ=255)`  
                                                                        `tgggctcggagatgtgtataagagacagcAACGATGCTTGTACTATTAACCCTTACGATGGCTGGGCGTGTCAAATTAGCTACATGGAGGAAACACCTGATGGCTCTTTGCGGCACCCATCGTTCGTAATGTTCCGTGGCACCGAGGACAACCCTCAAGAGAAAATGTAATCACACTGGCTCACCTTCGGGTGGGCCTTTCTGCGTTTATAAGGAGACACTTTATGTTTAAGAAGGTTGGTAAATTCCTTGCGGCTTTGGCAGCTATCCTGACGCTTGCGTATATTCTTGCGGTATACCCt                                                                                                                                                                                                                                      <  1:222328/273‑1 (MQ=255)`  
                                                                         `aTTCTTTAGCCCATACGGTATTGGCGACAACGATGCTTGTACTATTAACCCTTACGATGGCTGGGCGTGTCAAATTAGCTACATGGAGGAAACACATGATGGCTCTTTGCGGCACCCATCGTTCGTAATGTTCCGTGGCACCGAGGACAACCCTCAAGAGAAAATGTAATCACACTGGCTCACCTTCGGGTGGGCCTTTCTGCGTTTATAAGGAGACACTTTATGTTTAAGAAGGTTGGTAAATTCCTTGCGGCTTTGGCAGCTATCCTGACGCTTGCGTATATTCTTGCGGTATACCCTc                                                                                                                                                                                                                                     <  1:52353/301‑1 (MQ=255)`  
                                                                          `ttCTTTAGCCCATACGGTATTGGCGACAACGATGCTTGTACTATTAACCCTTACGATGGCTGGGCGTGTCAAATTAGCTACATGGAGGAAACACCTGATGGCTCTTTGCGGCACCCATCGTTCGTAATGTTCCGTGGCACCGAGGACAACCCTCAAGAGAAAATGTAATCACACTGGCTCACCTTCGGGTGGGCCTTTCTGCGTTTATAAGGAGACACTTTATGTTTAAGAAGATTGGTAAATTCCTTGCGGCTTTGGCAGCTATCCTGACGCTTGCGTATATTCTTCCGTTATACCCTCa                                                                                                                                                                                                                                    >  1:340635/1‑301 (MQ=255)`  
                                                                            `cTTTAGCCCATACGGTATTGGCGACAACGATGCTTGTACTATTAACCCTTACGATGGCTGGGCGTGTCAAATTAGCTACATGGAGGAAACACCTGATGGCTCTTTGCGGCACCCATCGTTCGTAATGTTCCGTGGCACCGAGGACAACCCTCAAGAGAAAATGTAATCACACTGGCTCACCTTCGGGTGGGCCTTTCTGCGTTTATAAGGAGACACTTTATGTTTAAGAAGGTTGGTAAATTCCTTGCGGCTTTGGCAGCTATCCTGACGCTTGCGTATATTCTTGCGGTATACCCTCAAg                                                                                                                                                                                                                                  >  1:83470/1‑301 (MQ=255)`  
                                                                             `tttAGCCCATACGGTATTGGCGACAACGATGCTTGTACTATTAACCCTTACGATGGCTGGGCGTGTCAAATTAGCTACATGGAGGAAACACCTGATGGCTCTTTGCGGCACCCATCGTTCGTAATGTTCCGTGGCACCGAGGACAACCCTCAAGAGAAAATGTAATCACACTGGCTCACCTTCGGGTGGGCCTTTCTGCGTTTATAAGGAGACACTTTATGTTTAAGAAGGTTGGTAAATTCCTTGCGGCTTTGGCAGCTATCCTGACGCTTGCGTATATTCTTGCGGTATACCCTCAAGt                                                                                                                                                                                                                                 <  1:26267/301‑1 (MQ=255)`  
                                                                                 `gCCCATACGGTATTGGCGACAACGATGCTTGTACTATTAACCCTTACGATGGCTGGGCGTGTCAAATTAGCTACATGGAGGAAACACCTGATGGCTCTTTGCGGCACCCATCGTTCGTAATGTTCCGTGGCACCGAGGACAACCCTCAAGAGAAAATGTAATCACACTGGCTCACCTTCGGGTGGGCCTTTCTGCGTTTATAAGGAGACACTTTATGTTTAAGAAGGTTGGTAAATTCCTTGCGGCTTTGGCAGCTATCCTGACGCTTGCGTATATTCTTGCGGGATACCCTCAAGTAGCa                                                                                                                                                                                                                             >  1:198731/1‑301 (MQ=255)`  
                                                                                 `agacagaCGGTATTGGCGACAACGATGCTTGTACTATTAACCCTTACGATGGCTGGGCGTGTCAAATTAGCTACATGGAGGAAACACCTGATGGCTCTTTGCGGCACCCATCGTTCGTAATGTTCCGTGGCACCGAGGACAACCCTCAAGAGAAAATGTAATCACACTGGCTCACCTTCGGGTGGGCCTTTCTGCGTTTATAAGGAGACACTTTATGTTTAAGAAGGTTGGTAAATTCCTTGCGGCTTTGGCAGCTATCCTGACGCTTGCGTATATTCTTGCGGTATACCCTCAAGTAGCa                                                                                                                                                                                                                             <  1:241449/295‑1 (MQ=255)`  
                                                                                  `cccATACGGTATTGGCGACAACGATGCTTGTACTATTAACCCTTACGATGGCTGGGCGTGTCAAATTAGCTACATGGAGGAAACACCTGATGGCTCTTTGCGGCACCCATCGTTCGTAATGTTCCGTGGCACCGAGGACAACCCTCAAGAGAAAATGTAATCACACTGGCTCACCTTCGGGTGGGCCTTTCTGCGTTTATAAGGAGACACTTTATGTTTAAGAAGGTTGGTAAATTCCTTGCGGCTTTGGCAGCTATCCTGACGCTTGCGTATATTCTTTCGGTATACCCTCAAGTAGCAc                                                                                                                                                                                                                            >  1:317453/1‑301 (MQ=255)`  
                                                                                  `cccATACGGTATTGGCGACAACGATGCTTGTACTATTAACCCTTACGATGGCTGGGCGTGTCAAATTAGCTACATGGAGGAAACACCTGATGGCTCTTTGCGGCACCCATCGTTCGTAATGTTCCGTGGCACCGAGGACAACCCTCAAGAGAAAATGTAATCACACTGGCTCACCTTCGGGTGGGCCTTTCTGCGTTTATAAGGAGACACTTTATGTTTAAGAAGGTTGGTAAATTCCTTGCGGCTTTGGCAGCTATCCTGACGCTTGCGTATATTCTTGCGGTATACCCTCAAGTAGCAc                                                                                                                                                                                                                            <  1:235797/301‑1 (MQ=255)`  
                                                                                          `gTATTGGCGACAACGATGCTTGTACTATTAACCCTTACGATGGCTGGGCGTGTCAAATTAGCTACATGGAGGAAACACCTGATGGCTCTTTGCGGCACCCATCGTTCGTAATGTTCCGTGGCACCGAGGACAACCCTCAAGAGAAAATGTAATCACACTGGCTCACCTTCGGGTGGGCCTTTCTGCGTTTATAAGGAGACACTTTATGTTTAAGCATGTTGGTAAATTCCTTGCGGCTTTGGCATCTATCCTGACGCTTGCGTATATTCTTTCGGTATACCCTCCAGTATCTCtattagta                                                                                                                                                                                                                    >  1:283411/1‑301 (MQ=255)`  
                                                                                          `gTATTGGCGACAACGATGCTTGTACTATTAACCCTTACGATGGCTGGGCGTGTCAAATTAGCTACATGGAGGAAACACCTGATGGCTCTTTGCGGCACCCATCGTTCGTAATGTTCCGTGGCACCGAGGACAACCCTCAAGAGAAAATGTAATCACACTGGCTCACCTTCGGGTGGGCCTTTCTGCGTTTATAAGGAGACACTTTATGGTTAAGCAGGTTGGTAAATTCCTTTCTGCTTTGGCAGCTATCCTGACGCTTGCGTATATTCTTGAGGTATACCCTCACGTAGCAAtagtatta                                                                                                                                                                                                                    >  1:126390/1‑301 (MQ=255)`  
                                                                                          `gTATTGGCGACAACGATGCTTGTACTATTAACCCTTACGATGGCTGGGCGTGTCAAATTAGCTACATGGAGGAAACACCTGATGGCTCTTTGCGGCACCCATCGTTCGTAATGTTCCGTGGCACCGAGGACAA‑CCTCAAGAGAAAATGTAATCACACTGGCTCACCTTCGGGTGGGCCTTTCTGCGTTTATAAGGAGACACTTTATGTTTAAGAAGGTTGGTAAATTCCTTGCGGCTTTTGCAGCTATCCTGACGCTTGCGTATATTCTTGCGGTATACCCTCAAGTAGCACtagtagtct                                                                                                                                                                                                                   >  1:166222/1‑299 (MQ=255)`  
                                                                                           `ataagagacagaCGATGCTTGTACTATGAACCCTTACGATGGCTGGGCGTGTCAAATTAGCTACATGGAGGAAACACCTGATGGCTCTTTGCGGCCACCATCGTTCGTAATGTTCCGTGGCACCGAGGACAACCCTCAAGAGAAAATGTAATCACACCGGCTCACCTTCGGGTGGGCCTTTCTGCGTTTATAAGGAGACACTTTATGTTTAAGAAGGTTGGTAAATTCCTTGCGGCTTTGGCAGCTATCCTGACGCTTGCGTATATTCTTGCGGTATACCCTCAAGTAGCACtagtagtag                                                                                                                                                                                                                   <  1:193077/290‑1 (MQ=255)`  
                                                                                            `tttGGCGAAAACGATGCTTGTACTATTAACCCTTACGATGGCTGGGCGTGTCAAATTAGCTACATGGAGGAAACACCTGATGGCTCTTTGCGGCACCCATCGTTCGTAATGTTCCGTGGCACCGAGGACAACCCTCAAGAGAAAATGTAATCACACTTGCTCACCTTCGGGTGGGCCTTTCTGCGTTTATAAGGAGACACTTTACGTTTAAGAAGGTTGGTAAATTCCTTGCGGCTTTGGCAGCTATCCTGACGCTTGCGTATATTCTTGCGGTATACCCTCAAGTAGCACTagtagtagt                                                                                                                                                                                                                  <  1:310145/300‑1 (MQ=255)`  
                                                                                            `cggagatgtgtaaaagagacagcTATTAACCCTTACGATGGCTGGGCGTGTCAAATTAGCTACATGGAGGCTACACCTGATGGCTCTTTGCGGCACCCATCGTTCGTAATGTTCCGTGGCACCGAGGACAACCCTCAAGAGAAAATGTAATCACACTGGCTCACCTTCGGGTGGGCCTTTCTGCGTTTATAAGGAGACACTTTATGTTTAAGAAGGTTGGTAAATTCCTTGCGGCTTTGGCAGCTATCCTGACGCTTGCGTATATTCTTGCGGTATACCCTCAAGTAGCACTagtagtagt                                                                                                                                                                                                                  <  1:73583/279‑1 (MQ=255)`  
                                                                                             `ttGGCGACAACGATGCTTGTACTATTAACCCTTACGATGGCTGGGCGTGTCAAATTAGCTACATGGAGGAAACACCTGATGGCTCTTTGCGGCACCCATCGTTCGTAATGTTCCGTGGCACCGAGGACAACCCTCAAGAGAAAATGTAATCACACTGGCTAACCTTCGGGTGGGCCTTTCTGCGTTTATAAGGAGACACTTTATGTTTAAGAAGGTTGGTAAATTCCTTGCGGCTTTGGCAGCTATCCTGACGCTTGCGTATATTCTTGCGGTATGCCCTCAAGTAGCACTAGTAGTAGtt                                                                                                                                                                                                                 <  1:337767/301‑1 (MQ=255)`  
                                                                                               `ggCGACAACGATGCTTGTACTATTAACCCTTACGATGGCTGGGCGTGTCAAATTAGCTACATGGAGGAAACACCTGATGGCTCTTTGCGGCACCCATCGTTCGTAATGTTCCGTGGCACCGAGGACAACCCTCAAGAGAAAATGTAATCACACTGGCTCACCTTCGGGTGGGCCTTTCTGCGTTTATAAGGAGACACTTTATGTTTAAGAAGGTTGGTAAATTCCTTGCGGCTTTGGCAGCTATCCTGACGCTTGCGTATATTCTTGCGGTATACCCTCAAGTAGCACTAGTAGTAGTTgg                                                                                                                                                                                                               <  1:100049/301‑1 (MQ=255)`  
                                                                                                 `cGACAACGATGCTTGTACTATTAACCCTTACGATGGCTGGGCGTGTCAAATTAGCTACATGGAGGAAACACCTGATGGCTCTTTGCGGCACCCATCGTTCGTAATGTTCCGTGGCACCGAGGACAACCCTCAAGAGAAAATGTAATCACACTGGCTCACCTTCGGGTGGGCCTTTCTGCGTTTATAAGGAGACACTTTATGTTTAAGAAGGTTGGTAAATTCCTTGCGGCTTTGGCAGCTATCCTGACGCTTGCGTATATTCTTGCGGTATACACTCAAGTAGCACTAGTAGTAGTTGgcg                                                                                                                                                                                                             <  1:16297/301‑1 (MQ=255)`  
                                                                                                      `aCGATGCTTGTACTATTAACCCTTACGATGGCTGGGCGTGTCAAATTAGCTACATGGAGGAAACACCTGATGGCTCTTTGCGGCACCCATCGTTCGTAATGTTCCGTGGCACCGAGGACAACCCTCAAGAGAAAATGTAATCACACTGGCTCACCTTCGGGTGGGCCTTTCTGCGTTTATAAGGAGACACTTTATGTTTAAGAAGGTTGGTAAATTCCTTGCGGCTTTGGCAGCTATCCTGACGCTTGCGTATATTCTTGCGGTATCCCCTCAAGTAGCACTAGTAGTAGTTGGCGCTTGt                                                                                                                                                                                                        >  1:109146/1‑301 (MQ=255)`  
                                                                                                          `tacTTGTACTATTAACCCTTACGATGGCTGGGCGTGTCAAATTAGCTACATGGAGGAAACACCTGATGGCTCTTTGCGGCACCCATCGTTCGTAATGTTCCGTGGCACCGAGGACAACCCTCAAGAGAAAATGTAATCACACTGGCTCACCTTCGGGTGGGCCTTTCTGCGTTTATAAGGAGACACTTTATGTTTAAGAAGGTTGGTAAATTCCTTGCGGATTTGGCAGCTATACTGACGCTTGCGTATATTCTTGCGGTATACCCTCAAGTAGCACTAGTAGTAGTTGGCGCTGGTTACt                                                                                                                                                                                                    >  1:135974/3‑301 (MQ=255)`  
                                                                                                          `tGCTTGTACTATTAACCCTTACGATGGCTGTGCGTGTCAAATTAGCTACATGGAGGAAACACCTGATGGCTCTTTGCGGCACCCATCGTTCGTAATGTTCCGTGGCACCGAGGACAACCCTCAAGAGAAAATGTAATCACACTGGCTCACCTTCGGGTGGGCCTTTCTGCGTTTATAAGGAGACACTTTATGTTTAAGAAGGTTGGTAAATTCCTTGCGGCTTTGGCAGCTATCCTGACGCTTGCGTATATTCTTGCGGTATCCCCTCAAGTAGCACTAGTAGTAGTTGGCGCTTGTTACt                                                                                                                                                                                                    >  1:172795/1‑301 (MQ=255)`  
                                                                                                          `tGCTTGTACTATTAACCCTTACGATGGCTGGGCGTGTCAAATTAGCTACATGGAGTAAACACCTGATGGCTCTTTGCGGCACCCATCGTTCGTAATGTTCCGTGGCACCGAGGACAACCCTCAAGAGAAAATGTAATCACACTGGCTCACCTTCGGGTGGGCCTTTCTGCGTTTATAAGGAGACACTTTATGTTTAAGAAGGTTGGTAAATTCCTTGCGGCTTTGGCAGCTATCCTGACGCTTGCGTATATTCTTGCGGTATACCCTCAAGTAGCACTAGTAGTAGttctgtctcttatac                                                                                                                                                                                                    >  1:170152/1‑288 (MQ=255)`  
                                                                                                          `tGCTTGTACTATTAACCCTTACGATGGCTGGGCGTGTCAAATTAGCTACATGGAGGAAACACCTGATGGCTCTTTGCGGCACCCATCGTTCGTAATGTTCCGTGGAACCGAGGACAACACTCAAGAGAAAATGTAATCACACTGGCTCCCCTTAGGGTGGGCATTTCTGCGTTTATAAGGAGACACTTTATGTTTAAGAAGGTTGGTAAATTCCTTGCGGCTTTGGCAGCTATCCTGACGCTTGCGTATATTCTTGCGGTATACCCTCACGTAGCACTAGTAGTAGTTGGCGCTTGTTACt                                                                                                                                                                                                    >  1:306592/1‑301 (MQ=255)`  
                                                                                                           `gCTTGTACTATTAACCCTTACGATGGCTGGGCTTGTCAAATTAGCTACATGGAAGAAACACCTGATGGCTCTTTGCGGCACCCATCGTTCGTAATGTTCCGTGGCACCGAGGACAACCCTCAAGAGAAAATGTAATCACACTGGCTCACCTTCGGGTGGGCCTTTCTGCGTTTATAAGGAGACACTTTATGTTTAAGAAGGTTGGTAAATTCCTTGCGGCTTTGGCAGCTATCCTGACGCTTGCGTATATTCTTGCGGTATACCCTCAAGTAGCACTAGTAGTAGTTGGCGCTTGTTACtt                                                                                                                                                                                                   <  1:216467/301‑1 (MQ=255)`  
                                                                                                           `gCTTGTACTATTAACCCTTACGATGGCTGGGCGTGTCAAATTAGCTACATGGAGGAAACACCTGATGGCTCTTTGCGGCACCCATCGTTCGTAATGTTCCGTGGCACCGAGGACAACCCTCAAGAGAAAATGTAATCACACTGGCTCACCTTCGGGTGGGCCTTTCTGCGTTTATAAGGAGACACTTTATGTTTAAGAAGGTTGGTAAATTCCTTGCGGCTTTGGCAGCTATCCTGACGCTTGCGTATATTCTTGCGGTATACCCTCAAGTAGCACTAGTAGTAGTTGGCGCTTGTTACtt                                                                                                                                                                                                   <  1:331133/301‑1 (MQ=255)`  
                                                                                                             `ttGTACTATTAACCCTTACGATGGCTGGGCGTGTCAAATTAGCTACATGGAGGAAACACCTGATGGCTCTTTGCGGCACCCATCGTTCGTAATGTTCCGTGGCACCGAGGACAACCCTCAAGAGAAAATGTAATCACACTGGCTCACCTTCGGGTGGGCCTTTCTGCGTTTATAAGGAGACACTTTATGTTTAAGAAGGTTGGTAAATTCCTTGCGGATTTGGCAGCTATCCTGACGCTTGCGTATATTCTTGCGGTATACCCTCAAGTAGCACTAGTAGTAGTTGGCGCTTGTTACTTAg                                                                                                                                                                                                 <  1:238923/301‑1 (MQ=255)`  
                                                                                                                 `aCTCTTAACCCTTACGATGGCTGGGCGTGTCAAATTAGCTACATGGAGGAAACACCTGATGGCTCTTTGCGGCACCCATCGTTCGTAATGTTCCGTGGCACCGAGGACAACCCTCAAGAGAAAATGTAATCACACTGGCTCACCTTCGGGTGGGCCTTTCTGCGTTTATAAGGAGACACTTTATGTTTAAGAAGGTTGGTAAATTCCTTGCGGCTTTGGCAGCTATCCTAACGCTTGCGTATATTCTTGCGGTATACCCTCAAGTAGCACTAGTAGTAGTTGGCGCTTGTTACTTAGCGGc                                                                                                                                                                                             >  1:49487/1‑301 (MQ=255)`  
                                                                                                                  `cTATTAACCCTTACGATGGCTGGGCGTGTCAAATTAGCTACATGGAGGAAACACCTGATGGCTCTTTGCGGCACCCATCGTTCGTAATGTTCCGTGGCACCGAGGACAACCCTCAATAGAAAATGTAATCACACTGGCTCACCTTCGGGTGGGCCTTTCTGCGTTTATAAGGAGACACTTTATGTTTAAGAAGGTTGGTAAATTCCTTGCGGCTTTGGCAGCTATCCTGACGCTTGCGTATATTCTTGCGGTATACCCTCAAGTAGCACTAGTAGTAGTTGGCGCTTGTTACTTAGCGGCa                                                                                                                                                                                            >  1:6716/1‑301 (MQ=255)`  
                                                                                                                  `cTATTAACCCTTACGATGGCTGGGCGTGTCAAATTAGCTACATGGAGGAAACACCTGATGGCTCTTTGCGGCACCCATAGTTCGTAATGTTCCGTGGCACCGAGGACAACCCTCAAGAGAAAATGTAATCACACTGGCTCACCTTCGGGTAGGCCTTTCTGCGTTTATAAGGAGACACTTTATGTTTAAGAAGGTTGGTCACTTCCTTGCGGCCTTGGCATCTATCCTGCAGCTTGCGTATCTTCTTTCAGTATACACTCAAGTCGCCCTAGTAGTAGTTGGCGCTTTTTACTTAGCGGCa                                                                                                                                                                                            >  1:146052/1‑301 (MQ=255)`  
                                                                                                                  `cTATTAACCCTTACGATGGCTGGGCGCGTCAAATTAGCTACATGGAGGAAACACCTGATGGCTCTTTGCGGCACCCATCGTTCGTAATGTTCCGTGGCACCGAGGACAACCCTCAAGAGAAAATGTAATCACACTGGCTCACCTTCGGGTGGGCCTTTCTGCGTTTATAAGGAGACACTTTATGTTTAAGAAGGTTGGTAAATTCCTTGCGGCCTTGGCAGCTATCCTGACGCTTGCGTATATCCTTGCGGTATACCCTCAAGTAGCACTAGCAGTAGTTGGCGCTTGTTACCTAGCGGcc                                                                                                                                                                                            >  1:255670/1‑300 (MQ=255)`  
                                                                                                                  `cTAATAACCCTTACGATGGCTGGGCGTGTCAAATTAGCTACATGGAGGAAACACCTGATGGCTATTTGCGGCACCCATCGTTCGTAATGTTCCGTGGCACCGAGGACAACCCTCAAGAGAGAATGTAATCACACTGGCTCACCTTCGGGTGGGCCTTTCTGCGTTTATAAGGAGACACTTTATGTTTAAGAAGGTTGGTATATTCCTTGCGGCTTTGGCAGCTATCCTGACGCTTGCGTATATTCTTGCGGTATATCCTCAAGTAGCACTCGTAGTAGTTGGCGCTTGTTACTTAGCGGcg                                                                                                                                                                                            >  1:227972/1‑300 (MQ=255)`  
                                                                                                                               `cGATGGCTGGGCGTGTCAAATTAGCTACATGGAGGAAACACCTGATGGCTCTTTGCGGCACCCATCGTTCGTAATGTTCCGTGGCACCGAGGACAACCCTCAAGAGAAAATGTAATCACACTGGCTCACCTTCGGGTGGGCCTTTCTGCGTTTATAAGGAGACACTTTATGTTTAAGAAGGTTGGTAAATTCCTTGCGGCTTTGGCAGCTATCCTGACGCTTGCGTATATTCTTGCGGTATACCCTCAAGTAGCACTAGTAGTAGTTGGCGCTTGTTACTTAGCGGCAGTGTGTGCTTGCg                                                                                                                                                                               <  1:148671/301‑1 (MQ=255)`  
                                                                                                                                   `ggCTGGGCGTGTCAAATTAGCTACATGGAGGAAACACCTGATGGCTCTTTGCGGCACCCATCGTTCGTAATGTTCCGTGGCACCGAGGACAACCCTCAAGAGAAAATGTAATCACACTGGCTCACCTTCGGGTGGGCCTTTCTGTGTTTATAAGGAGACACTTTATGTTTAAGAAGGTTGGTAAATTCCTTGCGGCTTTGGCAGCTATCCTGACGCTTGCGTATATTCTTGCGGTATACCCTCAAGTAGCACTAGTAGTAGTTGGCGCTTGTTACTTAGCGGAAgtgctgtctcttataca                                                                                                                                                                           >  1:114764/1‑287 (MQ=255)`  
                                                                                                                                   `ggCTGGGCGTGTCAAATTAGCTACATGGAGGAAACACCTGATGGCTCTTTGCGGCACCCATCGTTCGTAATGTTCCGTGGCACCGAGGACAACCATCAAGAGAAAATGTAATCACACTGGCTCACCTTCGGGTGGGCCTTTCTGCGTTTATAAGGAGACACTTTATGTTTAAGAAGGTTGGTAAATTCCTTGCGGCTTTGGCAGCTATCCTGACGCTTGCGTATATTCTTGCGGTATACCCTCAAGTAGCACTAGTAGTAGTTGGCGCTTGTTACTTAGCGGAAGTGTGTGCTTGCGtgtg                                                                                                                                                                           >  1:29824/1‑301 (MQ=255)`  
                                                                                                                                    `gCTGGGCTTGTCAAATTAGCTACATGGAGGAAACACCTGATGGCTCTTTGCGGCACCCATCGTTCGTAATGTTCCGTGGCACCGAGGACAACCCTCAAGAGAAAATGTAATCACACTGGCTCACCTTCGGGTGGGCCTTTCTGCGTTTATAAGGAGACACTTTATGTTTAAGAAGGTTGGTAAATTCCTTGCGGCTTTGGCAGCTATCCTTACGCTTGCGTATATTCTTGCGGTATACCCTCAAGTAGCACTAGTAGTAGTTGGCGCTTGTTACTTAGCGGCAGTGTGTGCTTGCGTGTgg                                                                                                                                                                          <  1:54387/301‑1 (MQ=255)`  
                                                                                                                                         `gCGTGTCAAATTAGCTACATGGAGGAAACACCTGATGGCTCTTTGCGGCACCCATCGTTCGTAATGTTCCGTGGCACCGAGGACAACCCTCAAGAGAAAATGTAATCACACTGGCTCACCTTCGGGTGGGCCTTTCTGCGTTTATAAGGAGACACTTTATGTTTAAGAAGGTTGGTAAATTCCTTGCGGCTTTGGCAGCTATCCTTACGCTTGCGTATATTCTTGCGGTATACCCTCAAGTAGCACTAGTAGTAGTTGGCGCTTGTTACTTAGCGGCAGTGTGTGCTTGCGTGTGGAGtat                                                                                                                                                                     <  1:103107/301‑1 (MQ=255)`  
                                                                                                                                            `tgtCAAATTAGCTACATGGAGGAAACACCTGATGGCTCTTTGCGGCACCCATCGTTCGTAATGTTCCGTGGCACCGAGGACAACCCTCAAGAGAAAATGTAATCACACTGGCTCACCTTCGGGTGGGCCTTTCTGCGTTTATAAGGAGACACTTTATGTTTAAGAAGGTTGGTAAATTCCTTGCGGCTTTGGCAGCTATCCTGACGCTTGCGTATATTCTTGCGGTATACCCTCAAGTAGCACTAGTAGTAGTTGGCGCTTGTTACTTAGCGGCAGTGTGTGCTTGCGTGTGGAGTATAGt                                                                                                                                                                  <  1:232799/301‑1 (MQ=255)`  
                                                                                                                                            `gttaaaaGTAGCTAAATGGAGGAAACACCTGATGGCTCTTTGCGGCACCCATCGTTCGTAATGTTCCGTGGCACCGAGGACAACCCTCAAGAGAAAATGTAATCACACTGGCTCACCTTCGGGTGGGCCTTTCTGCGTTTATAAGGAGACACTTTATGTTTAAGAAGGTTGGTAAATTCCTTGCGGCTTTGGCAGCTATCCTGACGCTTGCGTATATTCTTGCGGTATACCCTCAAGTAGCACTAGTAGTAGTTGGCGCTTGTTACTTAGCGGCAGTGTGTGCTTGCGTGTGGAGTATAGt                                                                                                                                                                  <  1:47277/297‑1 (MQ=255)`  
                                                                                                                                            `ggtCAAATTAGCTACATGGATGAAACACCTGATGGCTCTTTGCGGCACCAATCGTTCGTAATGTTCCGTGGCACCGAGGACAACCCTCAAGAGAAAATGTAATCACACTGGCTCACCTTCGGGTGGGCCTTTCTGCGTTTATAAGGAGACACTTTATGTTTAAGAAGGTTGGTAAATTCCTTGCGGCTTTGGCAGCTATCATGACGCTTGCGTATATTCTTGCGGTATACCCTCAAGTAGCACTAGTAGTAGTTGGCGCTTGTTACTTAGCGGCAGTGTGTGCTTGCGTGTGGAGTATAGt                                                                                                                                                                  <  1:315109/300‑1 (MQ=255)`  
                                                                                                                                              `tgtgtataagagacaggaggaAACACCTGATGGCTCTTTGCGGCACCCATCGTTCGTAATGTTCCGTGGCACCGAGGACAACCCTCAAGAGAAAATGTAATCACACTGGCTCACCTTCGGGTGGGGCTTTCTGCGTTTATAAGGAGACACTTTATGTTTAAGAAGGTTGGTAAATTCCTTGCGGCTTTGGCAGCTATCCTGACGCTTGCGTATATTCTTGCGGTATACCCTCAAGTAGCACTAGTAGTAGTTGGCGCTTGTTACTTAGCGGCAGTATGTGCTTGCGTGTGGAGTATAGTTa                                                                                                                                                                <  1:183659/286‑1 (MQ=255)`  
                                                                                                                                                 `aaTTAGCTACATGGAGGAAACACCTGATGGCTCTTTGCGGCACCCATCGTTCGTAATGTTCCGTGGCACCGAGGACAACCCTCAAGAGAAAATGTAATCACACTGGCTCACCTTCGGGTGGGCCTTTCTGCGTTTATAAGGAGACACTTTATGTTTAAGAAGGTTGGTAAATTCCTTGCGGCTTTGGCAGCTATCCTGACGCTTGCGTATATTCTTGCGGTATACCCTCAAGTAGCACTAGTAGTAGTTGGCGCTTGTTACTTAGCGGCAgtgtgtctgtctcttatacacatctccgagc                                                                                                                                                             >  1:89910/1‑276 (MQ=255)`  
                                                                                                                                                        `taaaTGGAGGAAACACCTGATGGCTCTTTGCGGCACCCATCGTTCGTAATGTTCCGTGGCACCGAGGACAACCCTCAAGAGAAAATGTAATCACACTGGCTCACCTTCGGGTGGGCCTTTCTGCGTTTATAAGGAGACACTTTATGTTTAAGAAGGTTGGTAAATTCCTTGCGGCTTTGGCAGCTATCCTGACGCTTGCGTATATTCTTGCGGTATACCCTCAAGTAGCACTAGTAGTAGTTGGCGCTTGTTACTTAGCGGCAGTGTGTGCTTGCGTGTGGAGTATAGTTAACTGGTAATa                                                                                                                                                      <  1:81032/298‑1 (MQ=255)`  
                                                                                                                                                        `tACATGGAGGAAACACCTGATGGCTCTTTGCGGCACCCATCGTTCGTAATGTTCCGTGGCACCGAGGACAACCCTCAAGAGAAAATGTAATCACACTGGCTCACCTTCGGGTGGGCCTTTCTGCGTTTATAAGGAGACACTTTATGTTTAAGAAGGTTGGTAAATTCCTTGCGGCTTTGGCAGCTATCCTGACGCTTGCGTATATTCTTGCGGTATACCCTCAAGTAGCACTAGTAGTAGTTGGCGCTTGTTACTTAGCGGCAGTGTGTGCTTGCGTGTGGAGTATAGTTAACTGGTAATa                                                                                                                                                      <  1:312651/301‑1 (MQ=255)`  
                                                                                                                                                              `gagtaAACACCTGATGGCTCTTTGCGGCACCCTTCGTTCGTAATGTTCCGTGGCACCGAGGACAACCCTCAAGGGAAAATGTAATCACACTGGCTCACCTTCGGGTGGGCCTTTCTGCGTTTATAAGGAGACACTTTATGTTTAAGAAGGTTGGTAAATTCCTTGCGGCTTTGGCAGCTATCCTGACGCTTACGTATATTCTTGCGGTATACCCTCAAGTAGCACTAGTAGTAGTTGGCGCTTGTTACTTAGCGGCAGTGTGTGCTTGCGTGTGGAGTATAGTTAACTGGTAATACAACTc                                                                                                                                                <  1:154793/301‑1 (MQ=255)`  
                                                                                                                                                                   `aaCACCTGATGGCTCTTTGCGGCACCCATCGTTCGTAATGTTCCGTGGCACCGAGGACAACCCTCAAGAGAAAATGTAATCACACTGGCTCACCTTCGGGTGGGCCTTTCTGCGTTTATAAGGAGACACTTTATGTTTAAGAAGGTTGGTAAATTCCTTGCGGTTTTGGCAGCTATCCTGACGCTTGCGTATATTCTTGCGGTATACCCTCAAGTAGCACTAGTAGTAGTTGGCGCTTGTTACTTAGTGGCAGTGTGTGCTTGCGTGTGGAGTATAGTTAACTGGTAATACGACTCACTaa                                                                                                                                           <  1:224824/301‑1 (MQ=255)`  
                                                                                                                                                                      `cccTGATGGCTCTTTGCGGCACCCATCGTTCGTAATGTTCCGTGGCACCGAGGACAACCCTCAAGAGAAAATGTAATCACACTGGCTCACCTTCGGGTGAGCCTTTCTGCGTTTATAAGGAGACACTTTATGTTTAAGAAGGTTGGTAAATTCCTTGCGGCTTTGGCAGCTATCCTGACGCTTGCGTATATTCTTGCGGTATACCCTCAAGTAGCACTAGTAGTAGTTGGCGCTTGTTACTTAGCGGCAGTGTGTGCTTGCGTGTGGAGTATAGTTAACTGGTAATACGACTCACTAAagg                                                                                                                                        <  1:321120/300‑1 (MQ=255)`  
                                                                                                                                                                      `acCTGATGGCTCTTTGCGGCACCCATCGTTCGTAATGTTCCGTGGCACCTAGGACAACCCTCAAGAGAAAATGTAATCACACTGGCTCACCTTCGGGTGGGCCTTTCTGCGTTTATAATGAGACACTTTATGTTTAAGAAGGTTGGTAAATTCCTTGCGGCTTTGGCAGCTATCCTGACGCTTGCGTATATTCTTGCGGTATACCCTCAAGTAGCACTAGTAGTAGTTGGCGCTTGTTACTTAGCGGCAGTGTGTGCTTGCGTGTGGAGTATAGTTAACTGGTAATACGACTCACTAAagg                                                                                                                                        <  1:56909/301‑1 (MQ=255)`  
                                                                                                                                                                       `ccTGATGGCTCTTTGCGGCACCCATCGTTCGTAATGTTCCGTGGCACCGAGGACAACCCTCAAGAGAAAATGTAATCACACTGGCTCACCTTCGGGTGGGCCTTTCTGCGTTTATAAGGAGACACTTTATGTTTAAGAAGGTTGGTAAATTCCTTGCGGCTTTGGCAGCTATCCTGACGCTTGCGTATATTCTTGCGGTATACCCTCAAGTAGCACTAGTAGTAGTTGGCGCTTGTTACTTAGCGGCAGTGTGTGCTTGCGTGTGGAGTATAGTTAACTGGTAATACGACTCACTAAagga                                                                                                                                       >  1:273010/1‑301 (MQ=255)`  
                                                                                                                                                                         `tGATGGCTCTTTGCGGCACCCATCGTTCGTAATGTTCCGTGGCACCGAGGACAACCCTAAAGAGAAAATGTAATCACACTGGCTCACCTTCGGGTGGGCCTTTCTGCGTTTATAAGGAGACACTTTATGTTTAAGAAGGTTGGTAAATTCCTTGCGGCTTTGGCAGCTATCCTGACGCTTGCGTATATTCTTGCGGTATACCCTCAAGTAGCACTAGTAGTAGTTGGCGCTTGTTACTTAGCGACAGTGTGTGCTTGCGTGTGGAGTATAGTTAACTGGTAATACGACTCACTAAaggagg                                                                                                                                     <  1:268579/301‑1 (MQ=255)`  
                                                                                                                                                                            `acagtctTTGCGGCACCCATCGTTCGTAATGTTCCGTGGCACCGAGGACAACCCTCAAGAGAAAATGTAATCACACTGGCTCACCTTCGGGTGGGCCTTTCTGCGTTTATAAGGAGACACTTTATGTTTAAGAAGGTTGGTAAATTCCTTGCGGCTTTGGCAGCTATCCTGACGCTTGCGTATATTCTTGCGGTATACCCTCAAGTAGCACTAGTAGTAGTTGGCGCTTGTTACTTAGCGGCAGTGTGTGCTTGCGTGTGGAGTATAGTTAACTGGTAATACGACTCACTAAAGGAGGTac                                                                                                                                  <  1:285830/297‑1 (MQ=255)`  
                                                                                                                                                                              `gttctTTGCGGCACCCATCGTTCGTAATGTTCCGTGGCACCGAGGACAACCCTCAAGAGAAAATGTAATCACACTGTCTCACCTTCGGGTGGGCCTTTCTGCGTTTATAAGGAGACACTTTATGTTTAAGAAGGTTGGTAAAGTCCTTGCGGCTTTGGCAGCTATCCTGACGCTTGCGTATATTCTTGCGGTATACCCTCAAGTAGCACTAGTAGTAGTTGGCGCTTGTTACTTAGCGGCAGTGTGTGCTTGCGTGTGGAGTATAGTTAACTGGTAATACGACTCACTAAAGGAGGTacac                                                                                                                                <  1:190431/299‑1 (MQ=255)`  
                                                                                                                                                                              `gCTCTTTGCGGCACCCATCGTTCGTAATGTTCCGTGGAACCGAGGACAACCCTCAAGAGAAAATGTAATCACACTGGCTCACCGTCGGGTGGGCCTTTCTGCGTTTATAAGGAGACACTTTATGTTTAAGAAGGTTGGTAAATTCCTTGCGGCTTTGGCAGCTATCCTGACGCTTGCGTATATTCTTGCGGTATACCCTCAAGTAGCACTAGTAGTAGTTGGCGCTTGTTACTTAGCGGCAGTGTGTGCTTGCGTGTGGAGTATAGTTAACTGGTAATACGACTCACTAAAGGAGGTacac                                                                                                                                <  1:86485/301‑1 (MQ=255)`  
                                                                                                                                                                                 `ctTTGCGGCACCCATCGTTCGTAATGTTCCGGGGCACCGAGGACAACCCTCAAGAGAAAATGTAATCACACTGGCTCACCTTCGGGTGGGCCTTTCTGCGTTTATAAGGAGACACTTTATGTTTAAGAAGGTTGGTAAACTCCTTGCGGCTTTGGCAGCTATCCTGACGCTTGCGTATATTCTTGCGGTATACCCTCAAGTAGCACTAGTAGTAGTTGGCGCTTGTTACTTAGCGGCAGTGTGTGCTTGCGTGTGGAGTATAGTTAACTGGTAATACGACTCACTAAaggagacactatat                                                                                                                             <  1:111756/301‑10 (MQ=255)`  
                                                                                                                                                                                 `ctTTGCGGCACCCATCGTTCGTAATGTGCCGGGGCACCGAGGACAACCATCAAGAGAAAATGTAATCACACTGGCTCACCTTCGGGTGGGCCTTTCTGCGTTTATAAGGAGACACTTTATGTTGAAGAAGGTTGGTAAATTCCTTGCGGCTTTGGCAGCTATCCTGACGCTTGCGTATATTCTTGCGGTATACCCTCAAGTAGCACTAGTAGTAGTTGGCGCTTGTTACTTAGCGGCAGTGTGTGCTTGCGTGTGGAGTATAGTTAACTGGTAATACGACTCACTAAAGGAGGTACACAcc                                                                                                                             <  1:195720/301‑1 (MQ=255)`  
                                                                                                                                                                                       `gtgggctcggagatgtgtataagagacagaCCGAGGACAACCCTCAAGAGAAAATGTAATCACACTGGCTCACCTTCGGGTGGGCCTTTCTGCGTTTATAAGGAGACACTTTATGTTTAAGAAGGTTGGTAAATTCCTTGCGGCTTTGGCAGCTATCCTGACGCTTGCGTATATTCTTTCGGTATACCCTCAAGTAGCACTAGTAGTAGTTGGCGCTTGTTACTTAGCGGCAGTGTGTGCTTGCGTGTGGAGTATAGTTAACTGGTAATACGACTCACTAAAGGAGGTACACACCatgatg                                                                                                                       <  1:40383/272‑1 (MQ=255)`  
                                                                                                                                                                                        `tcggagatgtgtataagagacaggTGGCACCGAGGACAACCCTCAAGAGAAAATGTAATCACACTGGCTCACCTTCGGGTGGGCCTTTCTGCGTTTATAAGGAGACACTTTATGTTTAAGAAGGTTGGTAAATTCCTTGCGGCTTTGGCAGCTATCCTGACGCTTGCGTATATTCTTGCGGTATACCCTCAAGTAGCACTAGTAGTAGTTGGCGCTTGTTACTTAGCGGCAGTGTGTGCTTGCGTGTGGAGTATAGTTAACTGGTAATACGACTCACTAAAGGAGGTACACACCATGATGt                                                                                                                      <  1:302036/278‑1 (MQ=255)`  
                                                                                                                                                                                              `aTCGTTCGTAATGTTCCGTGGCACCGAGGACAACCCTCAAGAGAAAATGTAATCACACTGGCTCACCTTCGGGTGGGCCTTTCTGCGTTTATAAGGAGACACTTTATGTTTAAGAAGGTTGGTAAATTCCTTGCGGCTTTGGCAGCTATCCTGACGCTTGCGTATATTCTTGCGGTATACCCTCAAGTAGCACTAGTAGTAGTTGGCGCTTGTTACTTAGCGGCAGTGTGTGCTTGCGTGTGGAGTATAGTTAACTGGTAATACGACTCACTAACGGAGGTACACACCATGATGTACTTaa                                                                                                                >  1:64690/1‑301 (MQ=255)`  
                                                                                                                                                                                                         `tGTTCCGTGGCACCGAGGACAACCCTCAAGAGAAAATGTAATCACACTGGCTCACCTTCTGGTGGGCCTTTCTGCGTTTATAAGGAGACACTTTATGTTTAAGAAGGTTGGTAAATTCCTTGCGGCTTTGGCAGCTATCCTGACGCTTGCGTATATTCTTGCGGTATACCCTCAAGTAGCACTAGTAGTAGTTGGCGCTTGTTACTTAGCGGCAGTGTGTGCTTGCGTGTGGAGTATAGTTAACTGGTAATACGACTCACTAAAGTAGGTACACACCATGATGTACTTAATGCCATTACTc                                                                                                     <  1:334373/301‑1 (MQ=255)`  
                                                                                                                                                                                                             `ccGTGGCACCGAGGACAACCCTCAAGAGAAAATGTAATCACACTGGCTCACCTTCGGGTGGGCCTTTCTGCGTTTATAAGGAGACACTTTATGTTTAAGAAGGTTGGTAAATTCCTTGCGGCTTTGGCAGCTATCCTAACGCTTGCGTATATTCTTGCGGTATACCCTCAAGTAGCACTAGTAGTAGTTGGCGCTTGTTACTTAGCGGCAGTGTGTGCTTGCGTGTGGAGTATAGTTAACTGGTAATACGACTCACTAAAGGAGGTACACACCATGATGTACttctgtctcttatacacat                                                                                                 >  1:138828/1‑284 (MQ=255)`  
                                                                                                                                                                                                               `gTGGCACCGAGGACAACCCTCAATAGAAAATGTAATCACACTGGCTCACCTTCGGGTGGGCCTTTCTGCGTTTATAAGGAGACACTTTATGTTTAAGCAGGTTGTTAAATTCCTTGCGGCTTTGGCAGCTATCCTGACGCTTGCGTATATTCTTGCGGTATACCCTCAAGTAGCACTAGTAGTAGTTGGCGCTTGTTACTTAGCGGCAGTGTGTGCTTGCGTGTGGAGTATAGTTAACTGGTAATACGACTCACTACAGGCGGTACACACCATGCTGTACTTAATGCCATTACTCATCGtt                                                                                               >  1:76390/1‑300 (MQ=255)`  
                                                                                                                                                                                                                  `gaaCCGAGGACAACCCTCAAGAGAAAATGTAATCACACTGGCTCACCTTCGGGTGGGCCTTTCTGCGTTTATAAGGAGACACTTTATGTTTAAGAAGGTTGGTAAATTCCTTGCTGCTTTGGCAGCTATCCTGACGCTTGCGTATATTCTTGCGGTATACCCTCAAGTAGCCCTAGTAGTAGTTGGCGCTTGTTACTTAGCGGCAGTGTGTGCTTGCGTGTGGAGTATAGTTAACTGGTAATACGACTCACTAAAGGAGGTACACACCATGATGTACTTAATGCCATTACTCATCGTCAtt                                                                                            >  1:213501/3‑301 (MQ=255)`  
                                                                                                                                                                                                                        `aGGACAACCCTCAAGAGAAAATGTAATCACACTGGCTCACCTTCGGGTGGGCCTTTCTGCGTTTATAAGGAGACACTTTATGTTTAAGAAGGTTGGTAAATTCCTTGCGGCTTTGGCAGCTATCCTGACGCTTGCGTATATTCTTGCGGTATACCCTCAAGTAGCACTAGTAGTAGTTGGCGCTTGTTACTTAGCGGCAGTGTGTGCTTGCGTGTGGAGTATAGTTAACTGGTAATACGACTCACTAAAGGAGGTACACACCATGATGTACTTAATGCCATTACTCATCGTCATTGTAGGa                                                                                      <  1:122611/301‑1 (MQ=255)`  
                                                                                                                                                                                                                         `ggACAACCCTCAAGAGAAAATGTAATCACACTGGCTCACCTTCGGGTGGGCCTTTCTGCGTTTATAAGGAGACACTTTATGTTTAAGAAGGTTGGTAAATTCCTTGCGGCTTTGGCAGCTATCCTGACGCTTGCGTATATTCTTGCGGTATACCCTCAAGTAGCACTAGTAGTAGTTGGCGCTTGTTACTTAGCGGCAGTGTGTGCTTGCGTGTGGAGTATAGTTAACTGGTAATACGACTCACTAAAGGAGGTACACACCATGATGTACTTAATGCCATTACTCATCGTCATTGTAGGAt                                                                                     >  1:129845/1‑301 (MQ=255)`  
                                                                                                                                                                                                                                `ccTCAAGAGAAAATGTAATCACACTGGATCACCTTCGGGTGGGCCTTTCTGCGTTTATAAGGAGACACTTTATGTTTAAGAAGGTTGGTAAATTCCTTGCGGCTTTGGCAGCTATCCTGACGCTTGCGTATATTCTTGCGGTATACCCTAAAGTAGCACTAGTAGTAGTTGGCGCTTGTTACTTAGCGGCAGTGTGTGCTTGCGTGTGGAGTATAGTTAACTGGTAATACGACTCACTAAAGGAGGTACACACCATGATGTACTTAATGCCATTACTCATCGTCATTgctgtctcttatac                                                                              >  1:265514/1‑288 (MQ=255)`  
                                                                                                                                                                                                                                           `aaTGTAATCACACTGGCTCACCTTCGGGTGGGCCTTTCTGCGTTTATAAGGAGACACTTTATGTTTAAGAAGGTTGGTAAATTCCTTGCGGCTTTGGCAGCTATCCTGACGCTTGCGTATATTCTTGCGGTATACCCTCAAGTAGCACTAGTAGTAGTTGGCGCTTGTTACTTAGCGGCAGTGTGTGCTTGCGTGTGGAGTATAGTTAACTGGTAATACGACTCACTAAAGGAGGTACACACCATGATGTACTTAATGCCATTACTCATCGTCATTGTAGGATGCCTTGCGCCCCACTGtt                                                                   >  1:70740/1‑300 (MQ=255)`  
                                                                                                                                                                                                                                            `aTGTAATCACACTGGCTCACCTTCGGGTGGGCCTTTCTGCGTTTATAAGGAGACACTTTATGTTTAAGAAGGTTGGTAAATTCCTTGCGGCTTTGGCAGCTATCCTGACGCTTGCGTATATTCTTGCGGTATACCCTCAAGTAGCACTAGTAGTAGTTGGCGCTTGTTACTTAGCGGCAGTGTGTGCTTGCGTGTGGAGTATAGTTAACTGGTAATACGACTCACTAAAGGAGGTACACACCATGATGTACTTAATGCCATTACTCATCGTCATTGTAGGATGCCTTGCGCTCCACTGTAg                                                                  <  1:36335/301‑1 (MQ=255)`  
                                                                                                                                                                                                                                               `tAATCATACTGGCTCACCTTCGGGTTGGCCTTTCTGCGTTTATAAGGAGACACTTTATGTTTAAGAAGGTTGGTAAATTCCTTGCGGCTTTGGCAGCTATCCTGACGCTTGCGTATATTCTTGCGGTATACCCTCAAGTAGCACTAGTAGTAGTTGGCGCTTGTTACTTAGCGGCAGTGTGTGCTTGCGTGTGGAGTATAGTTAACTGGTAATACGACTCACTAAAGGAGGTACACACCATGATGTACTTAATGCCATTACTCATCGTCATTGTAGGATGCCTTGCGCTCCACTGTAGCga                                                               <  1:178469/301‑1 (MQ=255)`  
                                                                                                                                                                                                                                                 `aTCACACTGGCTCACCTTCGGGTGGGCCGTTCTGCGTTTATAAGGATACACCTTATTTTTAAGAAGTTTGGTAAATTCCTTGCGTCTTTGGCAGCTATCCTGACGCTTGCGTATATTCTTTTGGTATACCCTCAAGTAGCACTAGTAGTAGTTGGCGCTTGTTACTTAGCGGCAGTGTGTGCTTGCGTGTGGAGTATAGTTAACTGGTAATACGACTCACTAAAGGAGGTACACACCATGATGTACTTAATGCCATTACTCATCGTCATTGTAGTATGCCTTGCGCTCCACTGTAGCgatg                                                             <  1:208745/301‑1 (MQ=255)`  
                                                                                                                                                                                                                                                       `cgggatagctttCTGGTGGGCATTTTTGCGTTTATAAGGTAACACATTATGTTTACGAAGGTTGGTAAATTCCATGCGGCTTTAGCAGCTATCCTGAGGCTTGCGTATATTCTTGCGGTATAGCCTCAAGTAGCACTAGTAGTAGTTGGCGCTTGTTACTTAGCGGCAGTGTGTGCTTGCGTGTGGAGTATAGTTAACTGGTAATACGACTCACTAAAGGAGGTACACACCATGATGTACTTAATGCCATTACTCATCGTCATTGTAGGATGCCTTGCGCTCCACTGTAGCGATAATGata                                                       <  1:219135/291‑1 (MQ=255)`  
                                                                                                                                                                                                                                                       `cTGGCTCACCTTCGGGTGGGCCTTTCTGCGTTTATAAGGAGACACTTTATGTTTAAGAAGGTTGGTAAATTCCTTGCGGCTTTGGCAGCTATCCTGACGCTTGCGTATATTCTTGCGGTATACCCTCAAGTAGCACTAGTAGTAGTTGGCGCTTGTTACTTAGCGGCAGTGTGTGCTTGCGTGTGGAGTATAGTTAACTGGTAATACGACTCACTAAAGGAGGTACACACCATGATGTACTTAATGTCATTACTCATCGTCATTGTAGGATGCCTTGCGCTCCACTGTAGCgatgatgatt                                                       >  1:119854/1‑300 (MQ=255)`  
                                                                                                                                                                                                                                                           `cTCACCTTCGGGTGGGCCTTTCTGCGTTTATAAGGAGACACTTTATGTTTAAGAAGGTTGGTAAATTCCTTGCGGCTTTGGCAGCTATCCTGACGCTTGCGTATATTCTTGCGGTATACCCTCAAGGAGCACTAGTAGTAGTTGGCGCTTGTTACTTAGCGGCAGTGTGTGCTTGCGTGTGGAGTATAGTTAACTGGTAATACGACTCACTAAAGGAGGTACACACCATGATGTACTTAATGCCATTACTCATCGTCATTGTAGGATGCCTTGCGCTCCACTGTAGCGATGATGATATGcc                                                   <  1:327952/301‑1 (MQ=255)`  
                                                                                                                                                                                                                                                               `gtcggagatgtgtataagagacagtataAGGAGACACTTTATGTTTAAGAAGGTTGGTAAATTCCTTGCGGCTTTGGCAGCTATCCTGACGCTTGCGTATATTCTTGCGGTATACACTCAAGTAGCACTAGTAGTAGTTGGCGCTTGTTACTTAGCGGCAGTGTGTGCTTGCGTGTGGAGTATAGTTAACTGGTAATACGACTCACTAAAGGAGGTACACACCATGATGTACTTAATGCCATTACCCATCGTCATTGTAGGATGCCTTGCGCTCCACTGTAGCGATGATGATATGCCAGAt                                               <  1:117791/277‑1 (MQ=255)`  
                                                                                                                                                                                                                                                               `ccTTCGGGTGGGCCTTTCTGCGTTTATAAGGAGACACTTTATGTTTAAGAAGGTTGGTAAATTCCTTGCGGCTTTGGCAGCTATCCTGACGCTTGCATAAATTCTTGCGGTATACCCTCAAGTAGCACTAGTAGTAGTTGGCGCTTGTTACTTAGCGGCAGTGTGTGCTTGCGTGTGGAGTATAGTTAACTGGTAATACGACTCACTAAAGGAGGTACACACCATGATGTACTTAATGCCATTACTCATCGTCATTGTAGGATGCCTTGCGCTCCACTGTAGCGATGATGATATGCCAGAt                                               >  1:60653/1‑301 (MQ=255)`  
                                                                                                                                                                                                                                                                  `tCGGGTGGGCCTTTCTGCGTTTATAAGGAGACACTTTATGTTTAAGAAGGTTGGTAAATTCCTTGCGGCTTTGGCAGCTATCCTGACGCTTGCGTATATTCTTGCGGTATACCCTCAAGTAGCACTAGTAGTAGTTGGCGCTTGTTACTTAGCGGCAGTGTGTGCTTGCGTGTGGAGTATAGTTAACTGGTAATACGACTCACTAAAGGAGGTACACACCATGATGTACTTAATGCCATTACTCATCGTCATTGTAGGATGCCTTGCGCTCCACTGTAGCGATGATGATATGCCAGATGGt                                            >  1:205435/1‑301 (MQ=255)`  
                                                                                                                                                                                                                                                                          `gCCTTTCTGCGTTTATAAGGAGACACTTTATGTTTAAGAAGGTTGGTAAATTCCTTGCGGCTTTGGCAGCTATCCTGACGCTTGCGTATATTCTTGCGGTATACCCTCAAGTAGCACTAGTAGTAGTTGGCGCTTGTTACTTAGCGGCAGTGTGTGCTTGCGTGTGGAGTATAGTTAACTGGTAATACGACTCACTAAAGGAGGTACACACCATGATGTACTTAATGCCATTACTCATCGTCATTGTAGGATGCCTTTCGCTCCACTGTAGCGATGATGATATGCCAGATGGTCACGCTTa                                    >  1:43990/1‑301 (MQ=255)`  
                                                                                                                                                                                                                                                                              `tccTGCGTTTATAAGGAGGCACTTTAGGTTTGAGAAGGTTGGTAAATTCCTTGCGGCTTTGGCAGCTATCCTGACGCTTGCGTATATTCTTGCGGTATACCCTCAAGTAGCACTAGTAGTAGTTGGCGCTTGTTACTTAGCGGCAGTGTGTGCTTGCGTGTGGAGTATAGTTAACTGGTAATACGACTCACTAAAGGAGGTACACACCATGATGTACTTAATGCCATTACTCATCGTCATTGTATGATGCCTTGCGCTCCACTGTAGCTATGATGATATGCCAGATGGTCACGCTTAATAc                                <  1:93269/299‑1 (MQ=255)`  
                                                                                                                                                                                                                                                                                 `tGCGTTAATAAGGAGATACTTTATGTGTAAGAAAGTTGGTAAATGCCTTGCGGCTTTGGCAGCTATCCTGACGCTTGCGTATATTCTTGCGGTATACCCTCAAGTAGAACTAGTAGTAGTTGGCGCTTGTTACTTAGCGGCAGTGTGTGCTTGCGTGTGGAGTATAGTTAACTGTTAATACGACTCACTAAAGGAGGTACACACCATGATGTACTTAATGCCATTACTCATCGTCATTGTAGGATGCCTTGCGCTCCACTGTAGCGATGATGATATGCCAGATGGTCACGCTTAATACGAc                             <  1:244752/301‑1 (MQ=255)`  
                                                                                                                                                                                                                                                                                             `gagaCACTTTATGTTTAAGAAGGTTGGTAAATTCCTTGCGGCTTTGGCAGCTATCCTGACGCTTGCGTATATTCTTGCGGTATACCCTCAAGTAGCACTAGTAGTAGTTGGCGCTTGTTACTTAGCGGCAGTGTGTGCTTGCGTGTGGAGTATAGTTAACTGGTAATACGACTCACTAAAGGAGGTACACACCATGATGTACTTAATGCCATTACTCATCGTCATTGTAGGATGCCTTGCGCTCCACTGTAGCGATGATGATATGCCAGATGGTCACGCTTAATACGACTCACTAAAGgag                 <  1:259829/301‑1 (MQ=255)`  
                                                                                                                                                                                                                                                                                                 `agacagaTGTTTAAGAAGGTTGGTAAATTCCTTGCGGCTTTGGCAGCTATCCTGACGCTTGCGTATATTCTTGCGGTATACCCTCAAGTAGAACTAGTAGTAGTTGGCGCTTGTTACTTAGCGGCAGTGTGTGCTTGCGTGTGGAGTATAGTTAACTGGTAATACGACTCACTAAAGGAGGTACACACCATGATGTACTTAATGCCATTACTCATCGTCATTGTAGGATGCATTGCGCTCCACTGTAGCGATGATGATATGCCAGATGGTCACGCTTAATACGACTCACTAAAGGAGacac             <  1:252887/295‑1 (MQ=255)`  
                                                                                                                                                                                                                                                                                                   `cTTTATGTTTAAGAAGGTTGGTAAATTCCTTGCGGCTTTGGCAGCTATCCTGACGCTTGCGTATATTCTTGCGGTATACCCTCAAGTAGCACTAGTAGTAGTTGGCGCTTGTTACTTAGCGGCAGTGTGTGCTTGCGTGTGGAGTATAGTTAACTGGTAATACGACTCACTAAAGGAGGTACACACCATGATGTACTTAATGCCATTACTCATCGTCATTGTAGGATGCCTTGCGCTCCACTGTAGCGATGATGATATGCCATATGGTCACGCTTAATActgtctcttatacacatctccg           >  1:230229/1‑280 (MQ=255)`  
                                                                                                                                                                                                                                                                                                       `aTGTTTAGGAAGGTTGGTAAAGTCCTTTCGGCTTTGGCAGCTATCCTGACGCTTGCGTATATTCTTTCGGTATAACCTCAAGTAGCACTAGTAGTAGTTGGCGCTTGTTACTTAGCGGCAGTGTGTGCTTGCGTGTGGAGTATAGTTAACTGGTAATACGACTCACTAAAGGAGGTACACAACATGATGTACTTAATGCCATTACTCATAGTCATTGTAGGATGCCTTGCGCTCCACTGTAGCGATGATGATATGCCAGATGGTCACGCTTAATACGACTCACTAAAGGAGACACTATATg       <  1:288759/301‑1 (MQ=255)`  
                                                                                                                                                                                                                                                                                                         `ttttAAGAAGGTTGGTAAATTCCTTGCGGCTTTGGCAGCTATCCTGAAGCTTGCGTATATTCTTGCTGTATACCCTCAAATAGCACTAGTAGTAGTTGGCGCTTGTTACTTAGCGGCAGTGTGTGCTTGCGTGTGGAGTATAGTTAACTGGTAATACGACTCACTAAAGGAGGTACACACCATGATGTACTTAATGCCATTACTCATCGTCATTGTAGGATGCCTTGCGCTCCACTGTAGCGATGATGATATGCCAGATGGTCACGCTTAATACGACTCACTAAAGGAGACACTATATGtt     <  1:294355/300‑1 (MQ=255)`  

                                                                                                                                                                                                                                                                                                            `|`                                                                                                                                                                                                                                                                                                              
`CCACGAATATCTCTCGCGCCTTAATGGATGAGTTCACTGAGACAGTAAAAGAGGCCACCCTAAGTCAATGGGGATTCTTTAGCCCATACGGTATTGGCGACAACGATGCTTGTACTATTAACCCTTACGATGGCTGGGCGTGTCAAATTAGCTACATGGAGGAAACACCTGATGGCTCTTTGCGGCACCCATCGTTCGTAATGTTCCGTGGCACCGAGGACAACCCTCAAGAGAAAATGTAATCACACTGGCTCACCTTCGGGTGGGCCTTTCTGCGTTTATAAGGAGACACTTTATGTTTAAGAAGGTTGGTAAATTCCTTGCGGCTTTGGCAGCTATCCTGACGCTTGCGTATATTCTTGCGGTATACCCTCAAGTAGCACTAGTAGTAGTTGGCGCTTGTTACTTAGCGGCAGTGTGTGCTTGCGTGTGGAGTATAGTTAACTGGTAATACGACTCACTAAAGGAGGTACACACCATGATGTACTTAATGCCATTACTCATCGTCATTGTAGGATGCCTTGCGCTCCACTGTAGCGATGATGATATGCCAGATGGTCACGCTTAATACGACTCACTAAAGGAGACACTATATGTTTCG  >  NC_001604.1/7313‑7913`

  

Alignment Legend

Aligned base mismatch/match (shaded by quality score): `ATCG/ATCG` `< 3 ≤` `ATCG/ATCG` `< 11 ≤` `ATCG/ATCG` `< 24 ≤` `ATCG/ATCG` `< 35 ≤` `ATCG/ATCG` `< 38 ≤` `ATCG/ATCG`

Unaligned base: `atcg`    Masked matching base: `atcg`    Alignment gap: `‑`    Deleted base: `‑`

Reads not counted as support for junction

read\_name Not counted due to insufficient overlap past the breakpoint.

read\_name Not counted due to not crossing MOB target site duplication.
